# Supplementary material for: DNA methylation landscape of ocular tissue relative to matched peripheral blood
Source: Sci Rep. 2017 Apr 13;7:46330. doi: 10.1038/srep46330 (PMC5390318; doi:10.1038/srep46330)
Supplement: Supplementary Information [file srep46330-s1.pdf]

Supplementary Information

DNA methylation landscape of ocular tissue relative to matched peripheral blood.

Alex W Hewitt
Vania Januar,
Alexandra Sexton-Oates,
Jihoon E Joo,
Maria Franchina,
Jie Jin Wang,
Helena Liang,
Jamie E Craig,
Richard Saffery

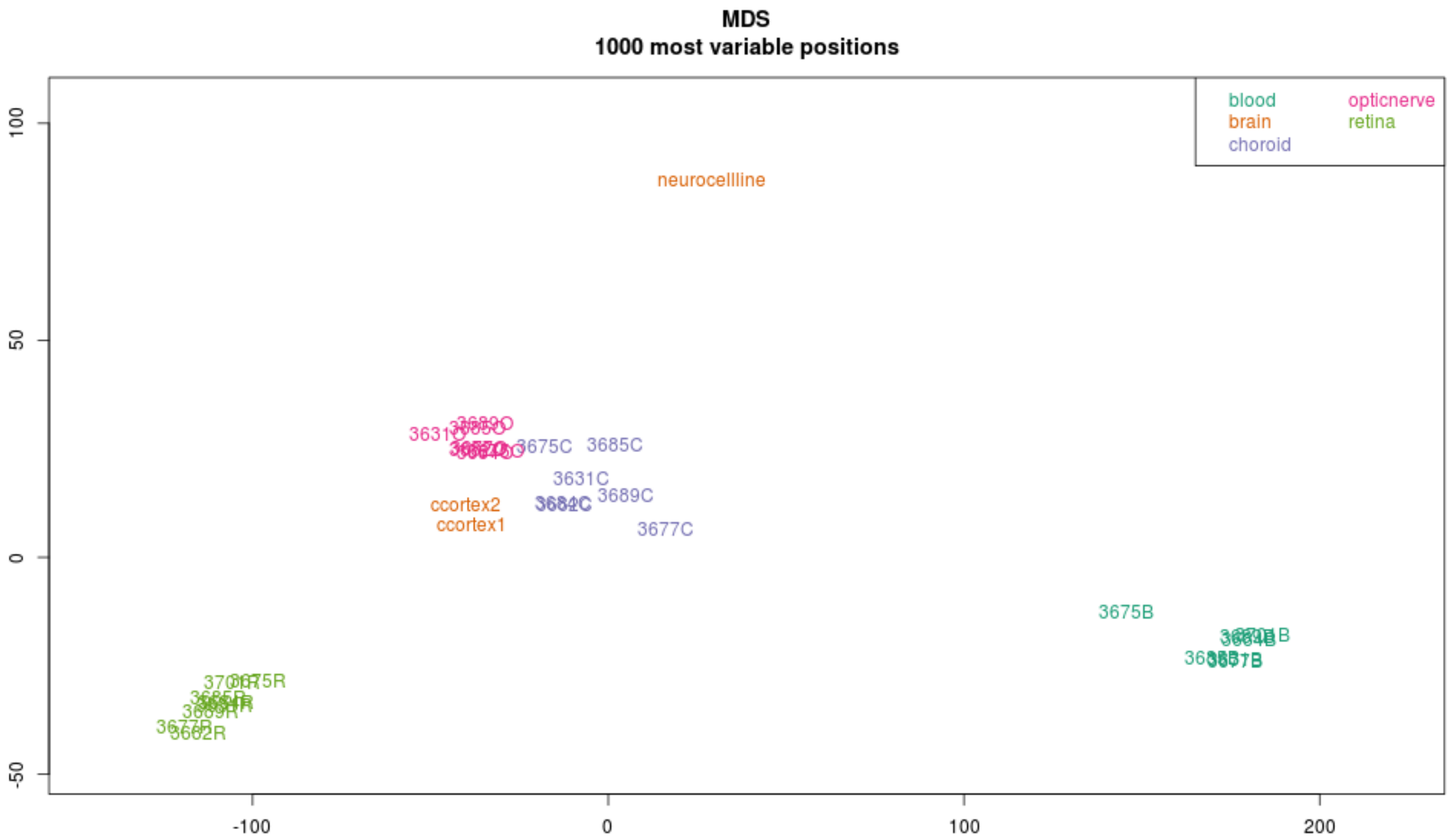

Supp Figure 1

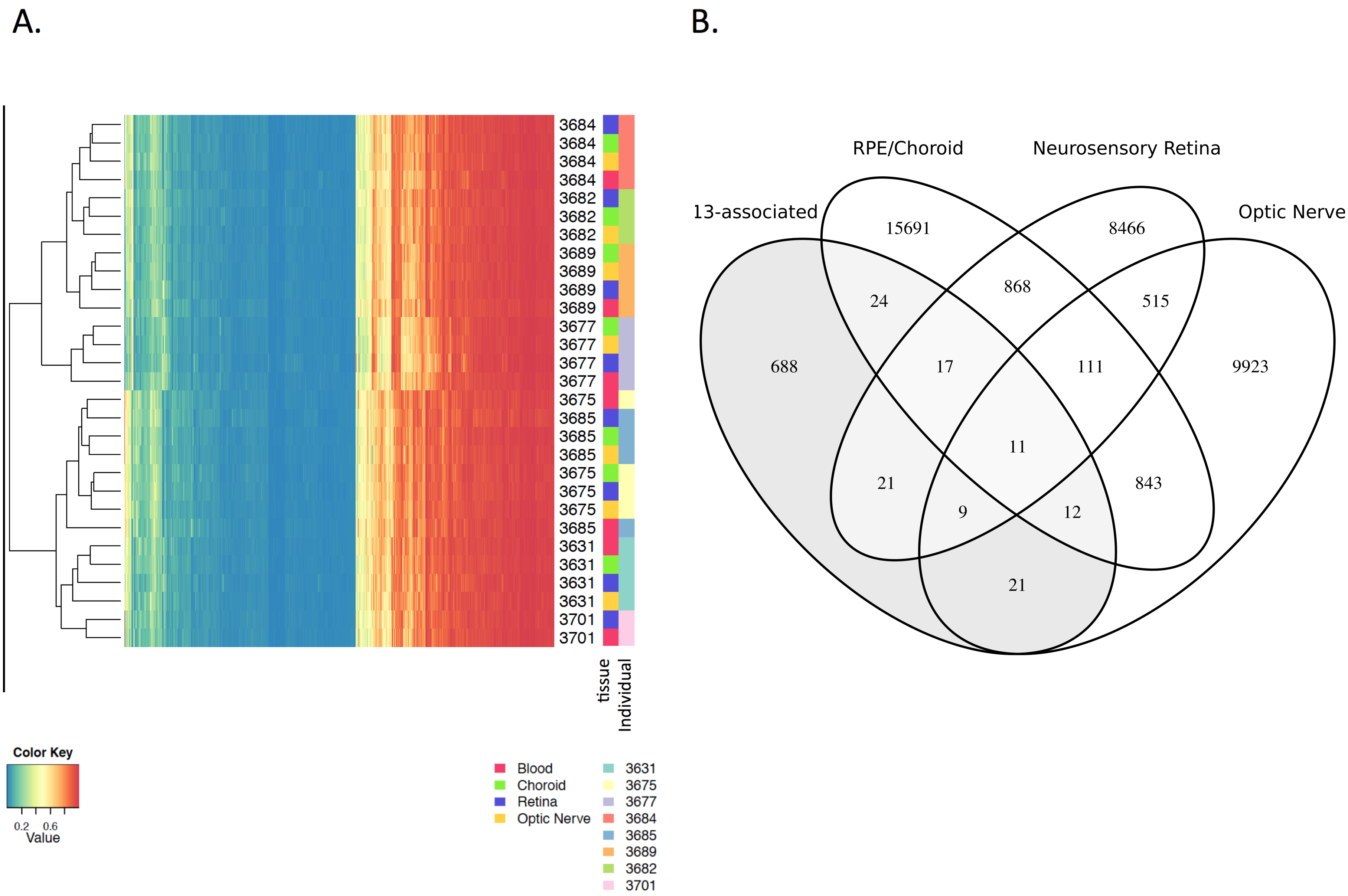

Supp Figure 2

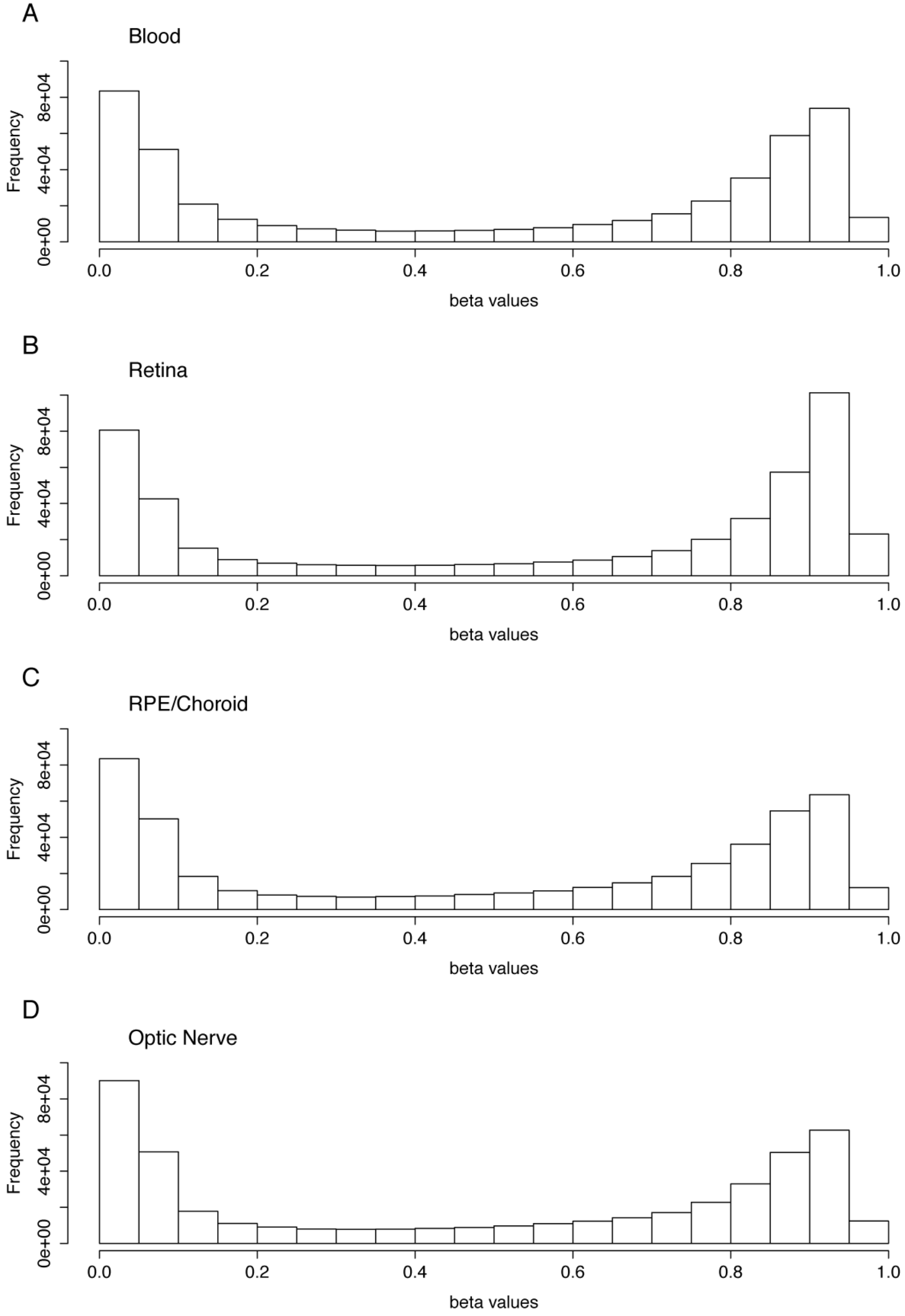

Supp Figure 3

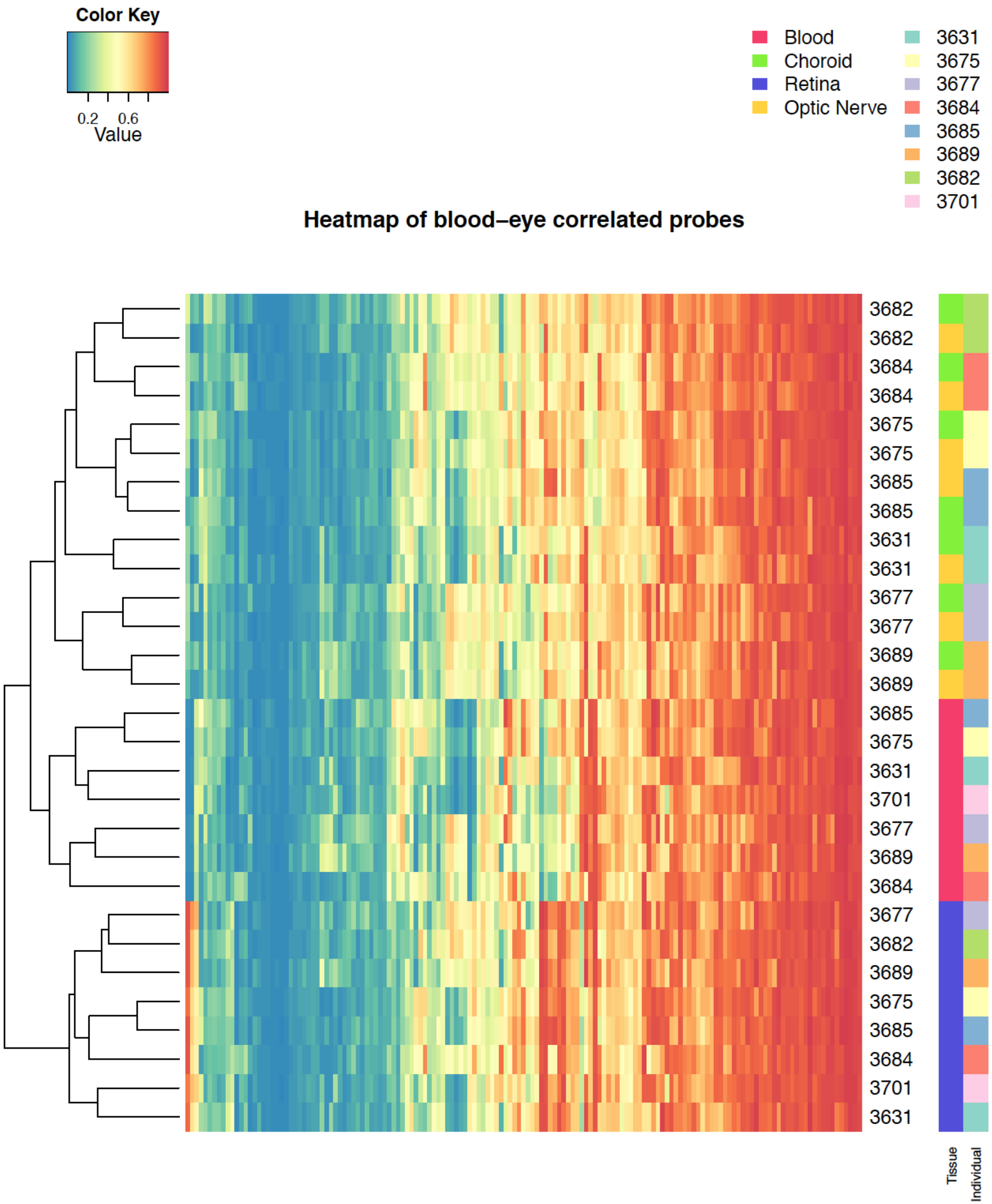

Supp Figure 4

Supplementary Figure 1. Multi Dimensional Scaling plot showing the relationship between samples based on 1000 most variable probes. Whereas RPE/choroid (C) and optic nerve (O) tissue cluster together, with methylation profiles similar to adult cortex tissue, retinal (R) methylation profile is distinct, with blood (B) being least similar to all other tissues. A neuronal cell line is included for comparative purposes.

Supplementary Figure 2. (A) Heatmap of probes associated with PC13 at $|r| > 0.5$, showing some clustering according to individual rather than tissue type. Blue denotes fully unmethylated (0 or 0% methylation), while red fully methylated (1 or 100% methylated) probes. (B) Venn diagram showing the overlap of PC13-associated probes with each of the 3 ocular tissues.

Supplementary Figure 3. Distribution of mean HM450K methylation b-values (in 5% bins) across each tissue tested in the current study. All tisseus show a bimodal distribution. X-axis b-values correspond to methylation levels approximating 0-100%. Y-axis is the frequency of probes within each bin.

Supplementary Figure 4.
Heatmap of 122 probes showing inter-individual variation in blood that show a degree of correlation between blood and all three eye tissues within individuals. Whereas clear inter-individual differences are apparent in the choroid and optic nerve, blood and retina remain clustered by tissue type. Blue denotes fully unmethylated (0 or 0% methylation), while red fully methylated (1 or 100% methylated) probes.

Supplementary Table 1.
Demographic details of samples included in analysis.

ID	Age at Death (years)	PTI (hours)	Cause of Death	Known Co-morbidities	Samples Passing QC			
					Whole Blood	Neurosensory Retina	Choroid/RPE	Optic Nerve
3684	37	3.1	Hanging	Depression, Asthma	X	X	X	X
3675	56	9.3	AMI	Type II Diabetes, Hypercholesterolaemia, Peptic Ulcer Disease	X	X	X	X
3631	58	8.2	AMI	Hypertension, Hypercholesterolaemia, Peptic Ulcer Disease	X	X	X	X
3685	63	11.3	AMI	Type II Diabetes,Hypertension, Hypercholesterolaemia, Asthma	X	X	X	X
3701	62	6.0	SAH	Type II Diabetes,Hypertension, Hypercholesterolaemia, Ischaemic Heart Disease	X	X		
3682	66	9.4	Respiratory Failure	Oesophogeal adenocarcinoma, Type II diabetes		X	X	X
3689	67	9.1	AMI	Prostate cancer	X	X	X	X
3677	76	10.1	AMI	Hypercholesterolaemia	X	X	X	X

Abbreviations: PTI, preservation time interval; AMI, Acute myocardial infarction; SAH, Subarachnoid haemorrhage.

Supplementary Table 3.

Genes linked to probes that variation exclusively associated with individuals at $p < 0.05$ (PC13), which have been previously implicated in eye disease.

Gene	Disease / Trait													Reference
	AMD	Age-related nuclear cataracts	Corneal curvature	PXF	Eye color	Glaucoma	Myopia (pathological)	Optic cup area	VCDR	Syndromic Retinal Dystrophy	RP	LCA	Recessive optic atrophy	
ACBD5										X				1
ADAMTS18										X				2,3
ASB7								X						4
B3GALTL	X													5,6
CACNA1A				X										7
CHEK2								X	X					4,8,9
CPLX2		X												10
DNAJC24						X								11
EIF2AK4			X											12
EXOC2									X					8
EXOSC2										X				13
FBXL17					X									14
GPR125											X			1
HARS										X				15
IQCB1										X		X		16,17
LRRK1			X											18
NOTCH4	X													19
PANK2										X				20-23
PDE2A								X						24
PTPRN2							X							25
TMEM126A													X	26
TOPORS											X			27
TRNT1										X				28,29
TUBGCP4										X				30
WFS1										X				31-33

Abbreviations: AMD, Age-related macular degeneration; PXF, Exfoliation syndrome; VCDR, Vertical cup-disc ratio; RP, Retinitis pigmentosa; LCA, Leber congenital amaurosis;

Supplementary References:

1 Abu-Safieh, L. *et al.* Autozygome-guided exome sequencing in retinal dystrophy patients reveals pathogenetic mutations and novel candidate disease genes. *Genome Res* **23**, 236-247, doi:10.1101/gr.144105.112 (2013).
2 Peluso, I. *et al.* The ADAMTS18 gene is responsible for autosomal recessive early onset severe retinal dystrophy. *Orphanet J Rare Dis* **8**, 16, doi:10.1186/1750-1172-8-16 (2013).
3 Aldahmesh, M. A. *et al.* Identification of ADAMTS18 as a gene mutated in Knobloch syndrome. *J Med Genet* **48**, 597-601, doi:10.1136/jmedgenet-2011-100306 (2011).
4 Springelkamp, H. *et al.* Meta-analysis of Genome-Wide Association Studies Identifies Novel Loci Associated With Optic Disc Morphology. *Genet Epidemiol* **39**, 207-216, doi:10.1002/gepi.21886 (2015).
5 Fritsche, L. G. *et al.* A large genome-wide association study of age-related macular degeneration highlights contributions of rare and common variants. *Nat Genet* **48**, 134-143, doi:10.1038/ng.3448 (2016).
6 Fritsche, L. G. *et al.* Seven new loci associated with age-related macular degeneration. *Nat Genet* **45**, 433-439, 439e431-432, doi:10.1038/ng.2578 (2013).
7 Aung, T. *et al.* A common variant mapping to CACNA1A is associated with susceptibility to exfoliation syndrome. *Nat Genet* **47**, 387-392, doi:10.1038/ng.3226 (2015).
8 Springelkamp, H. *et al.* Meta-analysis of genome-wide association studies identifies novel loci that influence cupping and the glaucomatous process. *Nat Commun* **5**, 4883, doi:10.1038/ncomms5883 (2014).
9 Ramdas, W. D. *et al.* A genome-wide association study of optic disc parameters. *PLoS Genet* **6**, e1000978, doi:10.1371/journal.pgen.1000978 (2010).
10 Liao, J. *et al.* Meta-analysis of genome-wide association studies in multiethnic Asians identifies two loci for age-related nuclear cataract. *Hum Mol Genet* **23**, 6119-6128, doi:10.1093/hmg/ddu315 (2014).
11 Hoffmann, T. J. *et al.* Genome-wide association and admixture analysis of glaucoma in the Women's Health Initiative. *Hum Mol Genet* **23**, 6634-6643, doi:10.1093/hmg/ddu364 (2014).
12 Mishra, A. *et al.* Genetic variants near PDGFRA are associated with corneal curvature in Australians. *Invest Ophthalmol Vis Sci* **53**, 7131-7136, doi:10.1167/iovs.12-10489 (2012).
13 Di Donato, N. *et al.* Mutations in EXOSC2 are associated with a novel syndrome characterised by retinitis pigmentosa, progressive hearing loss, premature ageing, short stature, mild intellectual disability and distinctive gestalt. *J Med Genet* **53**, 419-425, doi:10.1136/jmedgenet-2015-103511 (2016).
14 Candille, S. I. *et al.* Genome-wide association studies of quantitatively measured skin, hair, and eye pigmentation in four European populations. *PLoS One* **7**, e48294, doi:10.1371/journal.pone.0048294 (2012).
15 Puffenberger, E. G. *et al.* Genetic mapping and exome sequencing identify variants associated with five novel diseases. *PLoS One* **7**, e28936, doi:10.1371/journal.pone.0028936 (2012).
16 Otto, E. A. *et al.* Nephrocystin-5, a ciliary IQ domain protein, is mutated in Senior-Loken syndrome and interacts with RPGR and calmodulin. *Nat Genet* **37**, 282-288, doi:10.1038/ng1520 (2005).
17 Estrada-Cuzcano, A. *et al.* IQCB1 mutations in patients with leber congenital amaurosis. *Invest Ophthalmol Vis Sci* **52**, 834-839, doi:10.1167/iovs.10-5221 (2011).

18 Lu, Y. *et al.* Genome-wide association analyses identify multiple loci associated with central corneal thickness and keratoconus. *Nat Genet* **45**, 155-163, doi:10.1038/ng.2506 (2013).
19 Cipriani, V. *et al.* Genome-wide association study of age-related macular degeneration identifies associated variants in the TNXB-FKBPL-NOTCH4 region of chromosome 6p21.3. *Hum Mol Genet* **21**, 4138-4150, doi:10.1093/hmg/dds225 (2012).
20 Ching, K. H., Westaway, S. K., Gitschier, J., Higgins, J. J. & Hayflick, S. J. HARP syndrome is allelic with pantothenate kinase-associated neurodegeneration. *Neurology* **58**, 1673-1674 (2002).
21 Zhou, B. *et al.* A novel pantothenate kinase gene (PANK2) is defective in Hallervorden-Spatz syndrome. *Nat Genet* **28**, 345-349, doi:10.1038/ng572 (2001).
22 Hartig, M. B. *et al.* Genotypic and phenotypic spectrum of PANK2 mutations in patients with neurodegeneration with brain iron accumulation. *Ann Neurol* **59**, 248-256, doi:10.1002/ana.20771 (2006).
23 Hayflick, S. J. *et al.* Genetic, clinical, and radiographic delineation of Hallervorden-Spatz syndrome. *N Engl J Med* **348**, 33-40, doi:10.1056/NEJMoa020817 (2003).
24 Macgregor, S. *et al.* Genome-wide association identifies ATOH7 as a major gene determining human optic disc size. *Hum Mol Genet* **19**, 2716-2724, doi:10.1093/hmg/ddq144 (2010).
25 Meng, W. *et al.* A genome-wide association study provides evidence for association of chromosome 8p23 (MYP10) and 10q21.1 (MYP15) with high myopia in the French Population. *Invest Ophthalmol Vis Sci* **53**, 7983-7988, doi:10.1167/iovs.12-10409 (2012).
26 Hanein, S. *et al.* TMEM126A, encoding a mitochondrial protein, is mutated in autosomal-recessive nonsyndromic optic atrophy. *Am J Hum Genet* **84**, 493-498, doi:10.1016/j.ajhg.2009.03.003 (2009).
27 Chakarova, C. F. *et al.* Mutations in TOPORS cause autosomal dominant retinitis pigmentosa with perivascular retinal pigment epithelium atrophy. *Am J Hum Genet* **81**, 1098-1103, doi:10.1086/521953 (2007).
28 Chakraborty, P. K. *et al.* Mutations in TRNT1 cause congenital sideroblastic anemia with immunodeficiency, fevers, and developmental delay (SIFD). *Blood* **124**, 2867-2871, doi:10.1182/blood-2014-08-591370 (2014).
29 DeLuca, A. P. *et al.* Hypomorphic mutations in TRNT1 cause retinitis pigmentosa with erythrocytic microcytosis. *Hum Mol Genet* **25**, 44-56, doi:10.1093/hmg/ddv446 (2016).
30 Scheidecker, S. *et al.* Mutations in TUBGCP4 alter microtubule organization via the gamma-tubulin ring complex in autosomal-recessive microcephaly with chorioretinopathy. *Am J Hum Genet* **96**, 666-674, doi:10.1016/j.ajhg.2015.02.011 (2015).
31 Barrientos, A. *et al.* Autosomal recessive Wolfram syndrome associated with an 8.5-kb mtDNA single deletion. *Am J Hum Genet* **58**, 963-970 (1996).
32 Inoue, H. *et al.* A gene encoding a transmembrane protein is mutated in patients with diabetes mellitus and optic atrophy (Wolfram syndrome). *Nat Genet* **20**, 143-148, doi:10.1038/2441 (1998).
33 Young, T. L. *et al.* Non-syndromic progressive hearing loss DFNA38 is caused by heterozygous missense mutation in the Wolfram syndrome gene WFS1. *Hum Mol Genet* **10**, 2509-2514 (2001).
